# Characterization of *Bacillus velezensis* DY201: Antimicrobial Mechanisms and Intestinal Health Benefits in Broilers

**DOI:** 10.3390/ani16111677

**Published:** 2026-05-30

**Authors:** Yufei Liu, Shengmei Chen, Linlin Zhou, Qijing Zhang, Yufei Zhu, Wei Guo, Baoxia Ma, Shaona Jia, Xiaotao Ma, Xiaojun Yang, Kun Xu

**Affiliations:** 1 Hainan Institute of Northwest A&F University, Sanya 572024, China; liuyufei532@nwafu.edu.cn (Y.L.); 18208107626@163.com (S.C.); linlinzhou88@outlook.com (L.Z.); zqj15691195851@nwafu.edu.cn (Q.Z.);; 2 College of Animal Science and Technology, Northwest A&F University, Yangling 712100, China; 3 Shanxi Dayu Bioengineering Co., Ltd., Yuncheng 044000, China; 4 Jiangsu Institute of Poultry Science, Yangzhou 225125, China

**Keywords:** *Bacillus velezensis*, antimicrobial mechanisms, broiler intestinal health, multi-omics analysis

## Abstract

A novel *Bacillus velezensis* strain DY201, isolated from broiler feces, was characterized to assess its probiotic potential as an antibiotic alternative in poultry production. The strain demonstrated robust environmental tolerance with optimal growth at 42 °C and 51.32% survival following sequential exposure to simulated gastric and intestinal fluids. DY201 exhibited broad-spectrum antimicrobial activity against enterotoxigenic *Escherichia coli* K88, *Staphylococcus aureus*, *Salmonella pullorum*, and *Clostridium perfringens*, with activity remaining stable across pH 5.0–8.0 and retaining over 92.65% efficacy after 85 °C treatment. Scanning electron microscopy revealed metabolite-induced membrane perforation in target pathogens. Although whole-genome sequencing identified 14 biosynthetic gene clusters for lipopeptides including surfactin and fengycin, integrated proteomic and metabolomic analyses detected small-molecule metabolites—Withaferin A, 2′-hydroxy-2-methoxychalcone, and platycodigenin—as the primary antimicrobial effectors. In a preliminary broiler trial, dietary DY201 supplementation significantly increased the relative abundance of *Bacillus* in the ileum from 0.30% to 10.30% (*p* = 0.0434) and in the jejunum from 0.77% to 5.56% (*p* = 0.0453), enriched the generally beneficial genus *Lactobacillus* in the jejunum from 73.05% to 80.11% (*p* = 0.0323), and reduced *Candidatus Arthromitus* in the ileum from 13.38% to 0.59% (*p* = 0.0105). These findings support *B. velezensis* DY201 as a promising probiotic candidate for intestinal microbiota modulation in broilers, although functional intestinal health benefits require further validation through growth performance, barrier function, immune response, and pathogen challenge studies.

## Simple Summary

Reducing the use of antibiotics in poultry production requires safe and effective alternatives that can help control harmful bacteria and support intestinal health. In this study, we isolated a bacterial strain, *Bacillus velezensis* DY201, from broiler feces and evaluated its potential as a feed additive for chickens. This strain tolerated conditions similar to those in the chicken digestive tract and showed inhibitory activity against several important poultry-related harmful bacteria, including *Escherichia coli*, *Staphylococcus aureus*, *Salmonella pullorum*, and *Clostridium perfringens*. Its antibacterial activity remained relatively stable after heat treatment, changes in acidity, and simulated digestive conditions. Further analysis suggested that DY201 may inhibit harmful bacteria mainly by producing small compounds that damage bacterial cell surfaces. When included in the diet of broilers, DY201 changed the intestinal bacterial community in a moderate and site-specific manner, increasing some beneficial bacteria while reducing selected bacterial groups. These findings suggest that *Bacillus velezensis* DY201 has potential as a candidate feed additive for modulating the intestinal microbiota of broilers and may support intestinal health. However, functional health-related endpoints, including growth performance, intestinal morphology, barrier function, immune responses, and disease resistance, remain to be evaluated in future studies.

## 1. Introduction

As a primary source of animal protein, poultry contributes substantially to global food security. Contemporary farming systems prioritize sustainable practices, particularly interventions targeting broiler intestinal ecosystem optimization, a critical determinant of nutrient utilization and disease resistance [1]. The intestinal tract of broilers is a complex ecosystem that harbors a diverse microbiota, which has a significant impact on the broilers’ growth, digestion, immunity, and overall health.

Traditional approaches to maintaining intestinal health, such as the use of antibiotics, have become a cause for concern due to the emergence of antimicrobial-resistant (AMR) bacteria and the potential threats to public health. This has spurred the search for alternative strategies, including the use of organic acids, bacteriophages, and probiotics. While organic acids can reduce *Salmonella* colonization, they often impair feed palatability at effective doses [2]. Bacteriophages exhibit pathogen-specific suppression but require precise matching to farm-specific pathogen profiles [3]. In contrast, probiotics offer broader compatibility. Compared with other probiotics like *Lactobacillus* and *Bifidobacterium*, *Bacillus* have the advantages of stronger stress resistance and easier storage and transportation [4]. Among them, *Bacillus subtilis* has become an important probiotic [5,6]. Recently, a new member of the *Bacillus*, *Bacillus velezensis*, has also garnered increasing attention [7].

Accumulating evidence highlights *B. velezensis* as a multifunctional probiotic, with strain-specific capabilities ranging from antimicrobial compound synthesis (e.g., surfactin, iturin) to growth promotion via phytohormone modulation [8]. Previous studies have demonstrated that the average nucleotide identity (ANI) between *Bacillus methylotrophicus*, *Bacillus amyloliquefaciens* subsp. plantarum, *Bacillus oryzicola*, and *B. velezensis* exceeds 98%. They are classified as heterotypic synonyms [9]. Consequently, the name *B. velezensis* will be consistently used throughout this article. Previous studies have demonstrated that *B. velezensis* can produce a variety of antimicrobial substances, such as lipopeptides, bacteriocins, and organic acids, which can inhibit the growth of harmful bacteria and maintain the balance of the intestinal microbiota. For example, *B. velezensis* strain HeN-7 has been found to have strong antifungal activity and can produce a variety of antimicrobial substances, including surfactin, iturin, and fengycin [10]. *B. velezensis* strain 1273, for instance, produces surfactin homologues that inhibit Methicillin-resistant *Staphylococcus aureus* (MRSA) biofilm formation in vitro [11]. Moreover, *B. velezensis* can also enhance the immune function of animals by regulating the expression of immune-related genes and promoting the production of immune-related proteins [7]. Despite the well-documented antimicrobial potential of *B. velezensis*, most studies have focused on the characterization of its secondary metabolite gene clusters, particularly those encoding lipopeptides such as surfactin and fengycin, with the assumption that these are the primary effectors of antimicrobial activity. However, the actual metabolic output under specific culture conditions, and particularly its correlation with observed antimicrobial phenotypes, remains underexplored. Furthermore, while in vitro antimicrobial activity has been extensively reported, systematic evaluations of strain performance under conditions mimicking the gastrointestinal environment, as well as validation of in vivo efficacy in modulating gut microbiota and intestinal health, are limited.

In this study, we isolated and characterized a novel *B. velezensis* strain DY201, from broiler feces. Through a combination of phenotypic, genomic, proteomic, and metabolomic approaches, we systematically investigated its antimicrobial activity, environmental robustness, and underlying mechanisms. We demonstrate that DY201 exhibits potent, broad-spectrum antimicrobial activity against key poultry pathogens, including enterotoxigenic *Escherichia coli* K88 (ETEC K88), *Staphylococcus aureus* (*S. aureus*), *Salmonella pullorum* (*S. pullorum*), and *Clostridium perfringens* (*C. perfringens*), with remarkable stability under host physiological temperature, feed-processing heat, and simulated gastrointestinal conditions. Notably, multi-omics analysis revealed that while DY201 harbors a rich arsenal of lipopeptide biosynthetic gene clusters, its immediate antimicrobial activity under the tested conditions is primarily mediated by a distinct set of small-molecule metabolites, including Withaferin A and related compounds, rather than the anticipated lipopeptides. This unexpected finding challenges the prevailing assumption regarding the dominance of lipopeptides in *Bacillus* antimicrobial activity and highlights the metabolic versatility of this strain.

Moreover, in vivo evaluation in broilers demonstrated that dietary supplementation with DY201 significantly modulated gut microbiota composition in a segment-specific manner, increasing microbial diversity in the ileum, enriching beneficial genera such as *Lactobacillus*, and suppressing specific populations including *Candidatus Arthromitus*. These changes occurred without disrupting the overall community structure, indicating a “fine-tuning” mechanism characteristic of an ideal probiotic intervention. Collectively, our findings establish *B. velezensis* DY201 as a promising probiotic candidate for poultry production and provide novel insights into the complex, multi-layered antimicrobial strategies employed by this species, with implications for the development of effective antibiotic alternatives.

## 2. Results

### 2.1. Isolation, Identification, and Characterization of Bacillus velezensis Strain DY201

**Figure 1:**
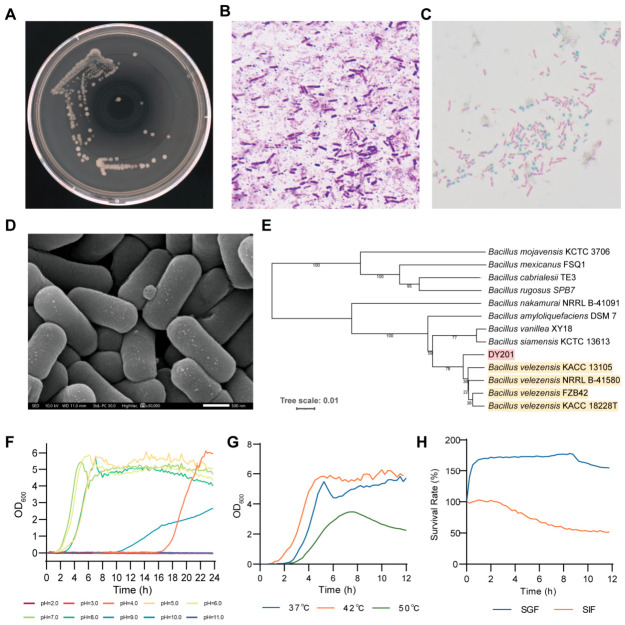
The morphological, genetic, and physiological characterization of *B. velezensis* strain DY201. (**A**) Macroscopic morphology on LB agar after 24 h incubation (37 °C). (**B**) Gram staining (1000×). (**C**) Endospore visualization via Schaeffer–Fulton staining: mature spores (green), vegetative cells (pink). (**D**) SEM micrograph (30,000×). (**E**) Phylogenetic tree based on whole-genome alignment with type strains. the pink highlight indicates *Bacillus velezensis* DY201, and the yellow highlight indicates the *B. velezensis* reference strains used for taxonomic comparison. (**F**) pH tolerance curves. (**G**) Thermotolerance curves. (**H**) Simulated gastrointestinal survival curves.

The novel strain DY201 was isolated from fecal samples of healthy Arbor Acres broilers collected by Shanxi Dayu Bioengineering Co., Ltd (Yuncheng, China). Colonies exhibited typical Bacillus morphology on LB agar, presenting as creamy white, circular formations with rough surfaces and irregular margins (Figure 1A). Gram staining confirmed the Gram-positive nature of DY201, with microscopic observation revealing rod-shaped vegetative cells and spores under malachite green staining (Figure 1B,C). Scanning electron microscopy (SEM) further resolved the ultrastructural features, showing smooth-surfaced bacilli with average dimensions of 1.41 μm × 0.61 μm (Figure 1D), consistent with the *B. velezensis* morphotype [8].

Biochemical characterization demonstrated DY201’s metabolic profile aligned with the *B. velezensis* taxon: positive for Voges–Proskauer (V-P) reaction, citrate utilization, and starch hydrolysis; negative for anaerobic growth and propionate utilization. Notably, the strain exhibited exceptional osmo-tolerance (7% NaCl) and acid resistance (pH 5.7), along with enzymatic activities including gelatin liquefaction and nitrate reduction, which are critical traits for intestinal persistence [12].

Whole-genome sequencing revealed a 4,176,678 bp circular chromosome (46.21% GC) and an 8118 bp plasmid (40.33% GC). Phylogenomic analysis based on whole-genome alignment positioned DY201 within the *B. velezensis* clade, sharing 97.69% ANI with the reference *B. velezensis* strain FZB42 (GCF_000015785.2) (Figure 1E), exceeding the 95% species delineation threshold [13].

Physiological profiling demonstrated DY201’s robust adaptability to poultry-relevant environments. Notably, the strain maintained sustained growth across a broad pH spectrum (4.0–9.0), This is a critical trait for surviving acidic stomach environments and alkaline intestinal environments (Figure 1F). Thermal tolerance assays further revealed active proliferation between 37–50 °C, with maximum growth rates observed at 42 °C, mirroring broiler core body temperature (Figure 1G). More importantly, gastrointestinal tolerance tests confirmed DY201’s functional resilience: When exposed to simulated gastric fluid (SGF), DY201 was able to replicate slowly. Subsequent intestinal persistence evaluation in simulated intestinal fluid (SIF) showed 51.32% survival retention despite trypsin stress, suggesting effective gastrointestinal tract colonization potential (Figure 1H).

The morphological consistency, genomic concordance, and environmental robustness confirm DY201 as a *B. velezensis* strain with potential probiotic applicability in poultry systems.

### 2.2. In Vitro Antimicrobial Activity and Stability of Bacillus velezensis Strain DY201

**Figure 2:**
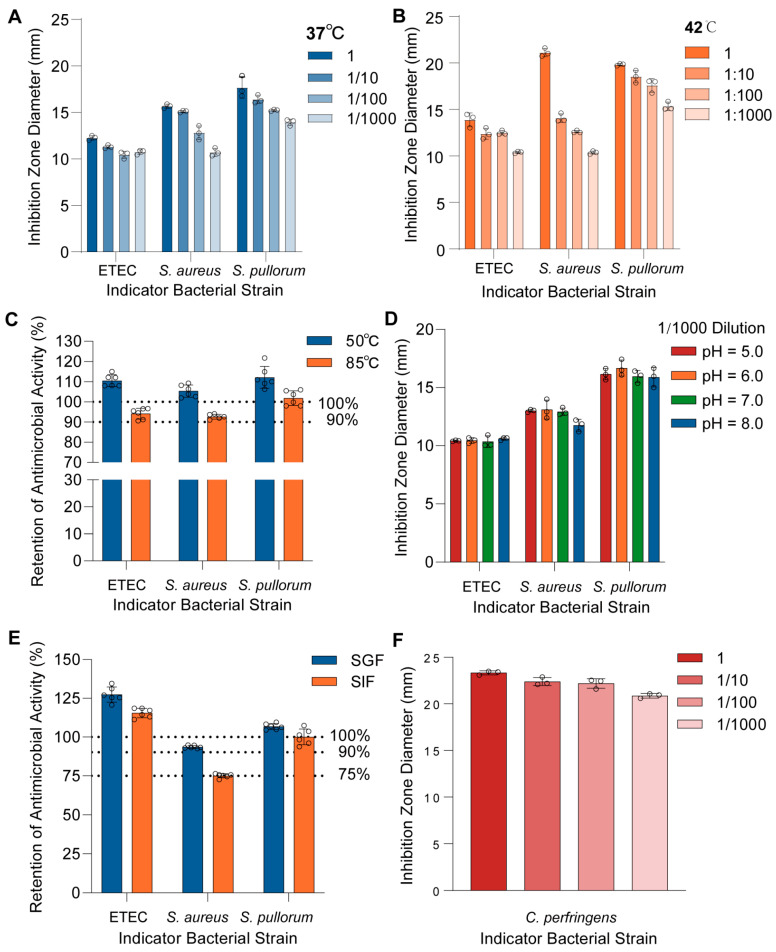
*Bacillus velezensis* strain DY201 exhibits broad-spectrum antimicrobial activity with high stability. (**A**) Inhibition of ETEC K88, *S. aureus*, and *S. pullorum* by DY201 supernatant. (**B**) Temperature effect. (**C**) Thermostability. (**D**) pH stability. (**E**) Gastrointestinal tolerance. (**F**) Inhibition of *C. perfringens* by DY201 supernatant. Data are presented as mean ± SD. Circles represent individual biological samples. The black dotted lines serve only as visual reference guides for the corresponding values and do not represent additional data points or statistical parameters.

Previous studies have indicated the potential of various *Bacillus* species to serve as alternatives to antibiotics [4,14]. To evaluate the application potential of the newly isolated strain *Bacillus velezensis* strain DY201, its in vitro antimicrobial activity was systematically assessed against three pathogens critically significant to livestock health: ETEC K88, *S. aureus*, and *S. pullorum*. The results demonstrated that DY201 exhibited strong inhibitory effects against *S. pullorum* and *S. aureus*, while a comparatively weaker, yet distinct, inhibitory activity was observed against ETEC K88 (Figure 2A). Notably, the cell-free supernatant of DY201 retained detectable antimicrobial activity even after a 1000-fold dilution. To simulate the in vivo environment within avian hosts, activity was evaluated at two typical body temperatures, 37 °C and 42 °C. Interestingly, the antimicrobial efficacy against all indicator strains, particularly against *S. pullorum* and *S. aureus*, was significantly enhanced at 42 °C compared to 37 °C, suggesting that its active metabolites function more effectively at the host’s physiological temperature (Figure 2B).

To assess its tolerance to feed pelleting processes, the supernatant was subjected to heat treatment at 50 °C and 85 °C for 5 min. Following 50 °C treatment, the antimicrobial activity was higher than that of the 37 °C control. After exposure to 85 °C, the activity showed a slight decrease compared to the 50 °C treatment; however, over 92.65% of the inhibitory activity was retained, confirming the exceptional thermostability of its active components and their suitability for enduring brief high-temperature feed processing (Figure 2C). Furthermore, DY201 displayed remarkable stability across a broad pH spectrum, as its antimicrobial activity remained largely unchanged when cultured in media with pH values ranging from 5.0 to 8.0 (Figure 2D). In tests simulating the gastrointestinal tract, treatment with SGF did not significantly alter the antimicrobial activity. Although a reduction was observed after exposure to SIF, substantial inhibitory activity was maintained, indicating that the active compounds can survive and remain functional within the complex intestinal environment (Figure 2E).

While ETEC K88, *S. pullorum*, and *S. aureus* are aerobic or facultative anaerobic bacteria, obligate anaerobes such as *C. perfringens* also pose a significant threat to livestock health. To further validate the broad-spectrum nature of DY201’s antimicrobial capacity, its activity against *C. perfringens* was evaluated under anaerobic conditions. The results revealed that DY201 effectively inhibited the growth of *C. perfringens*, producing an inhibition zone of 23.34 mm in diameter. Remarkably, this antimicrobial activity was largely preserved even after a 1000-fold dilution of the supernatant, demonstrating that the antimicrobial agents produced by DY201 remain efficacious under different oxygen conditions (Figure 2F).

In summary, *Bacillus velezensis* strain DY201 exhibits broad-spectrum and potent in vitro antimicrobial activity against several important livestock pathogens. This activity demonstrates outstanding stability under host physiological temperature, feed-processing heat, a wide pH range, and simulated gastrointestinal conditions, providing a solid in vitro foundation for its potential development as an antibiotic-alternative probiotic or feed additive.

### 2.3. From Phenotypic Observation to Molecular Mechanism: Multi-Omics Unravels the Antimicrobial Action of Bacillus velezensis Strain DY201

**Figure 3:**
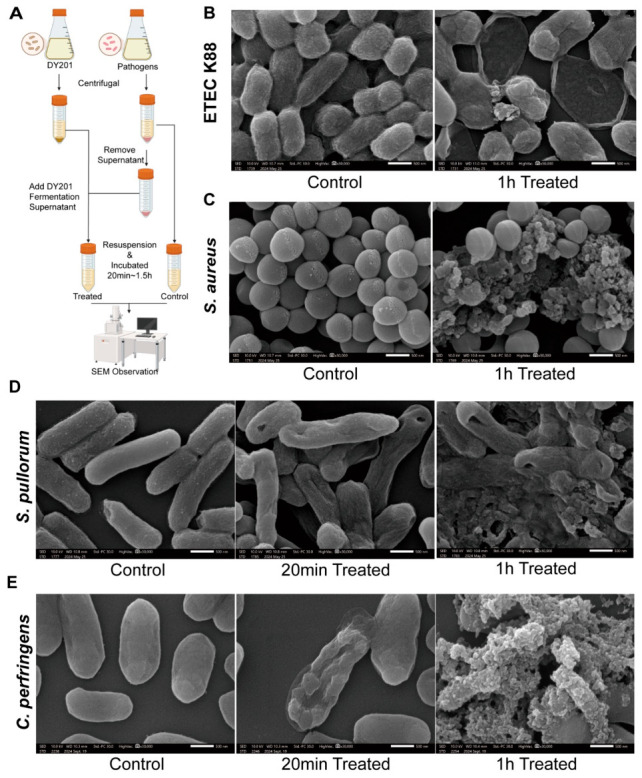
Scanning electron microscopy (SEM) reveals the membrane-targeting antimicrobial action of *Bacillus velezensis* strain DY201 metabolites. (**A**) Workflow. (**B**) ETEC K88. (**C**) *S. aureus*. (**D**) *S. pullorum*. (**E**) *C. perfringens*. Untreated controls display intact, smooth surfaces. Following exposure to DY201 supernatant, all pathogens show progressive membrane damage.

To elucidate the antimicrobial mechanism of *Bacillus velezensis* strain DY201, scanning electron microscopy (JEOL Ltd., Tokyo, Japan) was employed to examine the morphological alterations in pathogenic cells following treatment. The cell-free fermentation supernatant was used in this assay to unequivocally attribute any observed cellular damage to secreted metabolites, thereby avoiding ambiguity in distinguishing the DY201 cells from the target pathogens during microscopic analysis. The supernatant was co-incubated with each of the four indicator pathogens, ETEC K88, *S. aureus*, *S. pullorum*, and *C. perfringens*, under their optimal culture conditions for 20 min to 1.5 hours prior to fixation (Figure 3A).

As shown in Figure 3B–E, in stark contrast to the smooth, intact, and turgid cells in the untreated control, all pathogens exposed to the DY201 supernatant exhibited profound morphological damage. The treated cells displayed severe and consistent ultrastructural alterations, predominantly characterized by apparent depressions, pores, and fissures on the cell envelope. Extensive cell shrinkage and deformation were common, and complete membrane rupture was frequently observed, resulting in substantial leakage of intracellular contents and the presence of cellular debris. This pervasive compromise of cellular integrity across all four diverse pathogens demonstrates that the antimicrobial metabolites secreted by DY201 share a common, membrane-targeting mode of action.

**Figure 4:**
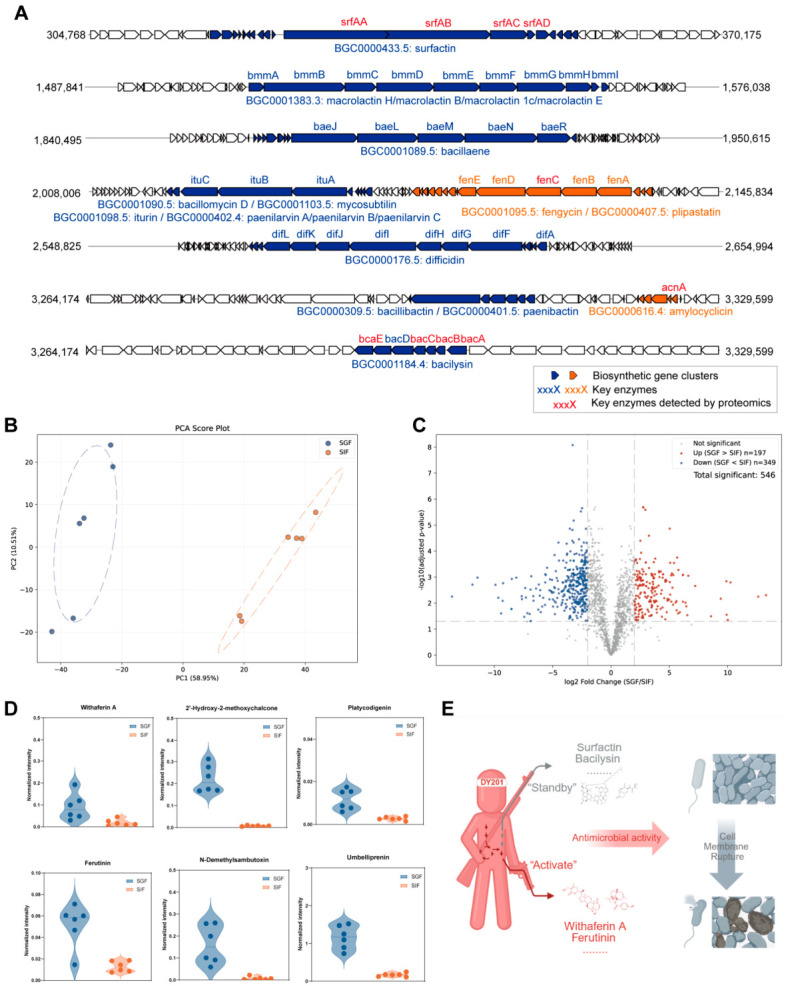
Multi-omics analysis of DY201 antimicrobial mechanism. (**A**) BGCs identified in the DY201 genome. (**B**) PCA score plot showing metabolic profile separation between the SGF group and the SIF group. (**C**) Metabolites differentially abundant between groups. (**D**) Relative abundance of key metabolites. (**E**) Proposed mechanism model.

Based on the established understanding that the primary antimicrobial mechanism of *Bacillus* species often involves lipopeptides or polyketides synthesized by nonribosomal peptide synthetase (NRPS) and polyketide synthase (PKS) gene clusters, a systematic genomic mining of the DY201 strain was conducted. Using antiSMASH 7.0, a total of 14 putative biosynthetic gene clusters (BGCs) with high similarity (82% to 100%) to known secondary metabolite clusters were identified (Figure 4A; Supplementary Table S1). The BGCs in DY201 were predominantly affiliated with the NRPS and PKS families, consistent with the typical genetic repertoire of bioactive *Bacillus* strains. Notably, 10 of the 14 clusters contained NRPS-related genes, and 6 contained PKS-related genes, establishing a strong genetic foundation for the production of complex antimicrobial compounds.

Further analysis identified key lipopeptide BGCs with high similarity to those encoding surfactin, iturin, bacillomycin D, mycosubtilin, and fengycin. Although the surfactin and iturin clusters showed some sequence divergence, they retained essential biosynthesis genes (*srfAA-AD* and *fenA-E*). Strikingly, DY201 harbored a 100% identical BGC for paenilarvin, an iturin-like lipopeptide with known antifungal and anticancer activities. This cluster, previously reported mainly in *Paenibacillus larvae*, highlights the unique metabolic potential of DY201.

The genome also contains BGCs for the iron-chelating siderophores bacillibactin and paenibactin, and a complete, conserved cluster for the polyketide difficidin. The presence of the key gene *dfnD* suggests potential production of this broad-spectrum compound, which inhibits protein synthesis and disrupts cell membranes. Additionally, a complete gene cluster for the circular bacteriocin amylocyclicin (involving *acnA-F* genes) was identified.

Beyond these major BGCs, an open reading frame encoding the bacteriocin LCI (UniProt: P82243) was identified at genomic position 299,877–300,161. Previous studies indicate LCI has modest antifungal but limited antimicrobial activity; thus, it likely does not contribute primarily to the observed membrane damage. In summary, the genome of *B. velezensis* DY201 harbors a rich array of NRPS, PKS, and other antimicrobial BGCs, providing a solid genetic basis for its significant in vitro antimicrobial activity.

To delineate the functional link between genetic potential and metabolic output, the fermentation supernatant of DY201 was subjected to integrated proteomic and non-targeted metabolomic analyses. Proteomic profiling identified several enzymes involved in the biosynthesis of canonical antimicrobial peptides typical of *Bacillus velezensis*, including those responsible for surfactin and iturin production. This indicates that the corresponding biosynthetic gene clusters were transcriptionally active and translationally engaged under the experimental conditions. However, neither the proteomic nor the metabolomic data detected the mature forms of these antimicrobial peptides (Figure 4A). These observations suggest that, under the current culture conditions, the expression levels of these canonical peptides may fall below the limit of detection, and that the antimicrobial activity of DY201 likely arises from alternative metabolites.

To identify metabolites directly contributing to antimicrobial efficacy, a differential metabolomic analysis was performed by comparing two treatment groups with markedly distinct antimicrobial potencies: the SGF-treated group (high activity) and the SIF-treated group (low activity). Principal component analysis (PCA) revealed a clear separation between the two groups (Figure 4B), indicating systematic differences in their metabolic profiles. Using the criteria of log_2_FC > 2 and *p* < 0.05, a total of 197 metabolites significantly upregulated in the SGF group were identified (Figure 4C). Among these, Withaferin A, 2’-Hydroxy-2-methoxychalcone, and Platycodigenin have been previously documented to disrupt bacterial cell membranes through mechanisms such as destabilization of the lipid bilayer, inhibition of biofilm formation, or induction of membrane pores, ultimately leading to leakage of intracellular contents (Figure 4D) [15,16,17]. This mode of action is fully consistent with the membrane perforation and lysis observed in our scanning electron microscopy analysis. In addition, several metabolites with reported antimicrobial activity but unresolved mechanisms, including Ferutinin, Umbelliprenin, and N-Demethylsambutoxin were also identified, offering promising candidates for further investigation [18,19,20].

Based on these multi-omics findings, we propose an integrated model for the antimicrobial mechanism of DY201 (Figure 4E). Under the experimental conditions employed in this study, DY201 employs metabolites such as Withaferin A as primary effector molecules for its immediate antimicrobial activity. Concurrently, the strain retains a complete set of gene clusters encoding various lipopeptide antibiotics and maintains constitutive expression of their key biosynthetic enzymes. These results demonstrate that DY201 possesses two coexisting antimicrobial systems: one comprising small-molecule metabolites actively produced under the current conditions, and another consisting of a lipopeptide biosynthetic pathway maintained in a “standby” state. The latter may be activated in response to specific environmental cues or host factors, thereby further enhancing the antimicrobial efficacy of the strain.

### 2.4. DY201 Modulates Gut Microbiota Composition and Diversity in a Segment-Specific Manner

**Figure 5:**
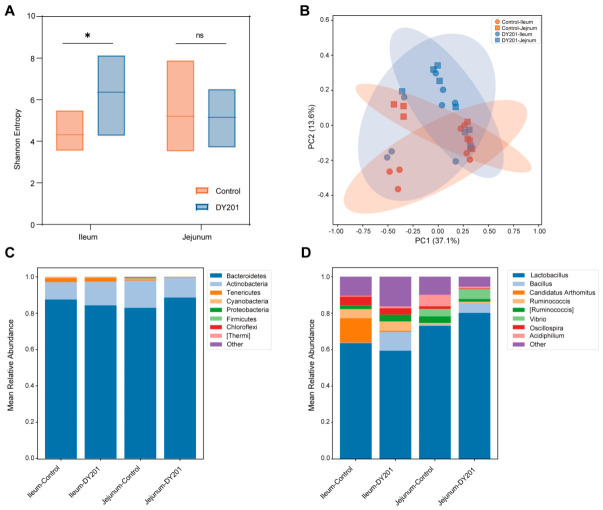
DY201 modulates gut microbiota in a segment-specific manner. (**A**) Alpha diversity. (**B**) Beta diversity. (**C**) Phylum-level changes. (**D**) Genus-level changes. For microbiota analysis, *n* = 8 birds per group. *, *p* < 0.05; ns, not significant.

To investigate the impact of DY201 supplementation on intestinal microecology, 16S rRNA gene sequencing was performed on luminal contents from the ileum and jejunum. Alpha diversity analysis revealed that DY201 supplementation significantly increased the Shannon entropy in the ileum (*p* < 0.05), indicating enhanced microbial richness and evenness in this segment. However, no significant difference in Shannon entropy was observed in the jejunum, suggesting that the effect of DY201 on microbial diversity is segment-specific (Figure 5A). Beta diversity analysis based on Bray–Curtis distances showed no significant separation between the control and DY201 groups, indicating that DY201 did not fundamentally disrupt the overall structure of the gut microbial community (Figure 5B).

Despite this overall structural stability, significant shifts were observed in specific bacterial taxa. At the phylum level, DY201 supplementation induced distinct changes in the two intestinal segments (Figure 5C; Supplementary Table S2). In the ileum, the relative abundance of Bacteroidetes increased approximately 29-fold compared to the control group, while in the jejunum, Firmicutes abundance increased from 82.90% to 88.59%.

At the genus level, marked shifts were observed in key bacterial taxa (Figure 5D; Supplementary Table S3). Most notably, the abundance of *Bacillus*, the genus to which DY201 belongs, increased dramatically in both intestinal segments, rising from 0.30% to 10.30% in the ileum (34-fold) and from 0.77% to 5.56% in the jejunum (7-fold). This confirms the successful colonization or transient survival of the supplemented strain in the chicken gut.

Interestingly, DY201 supplementation significantly enriched the beneficial genus *Lactobacillus* in the jejunum, with its relative abundance increasing from 73.05% in the control group to 80.11% in the DY201 group (*p* < 0.05). Conversely, the abundance of *Candidatus Arthromitus* in the ileum was markedly reduced from 13.38% to 0.59% following DY201 treatment. Other genera, including *Ruminococcus*, *Vibrio*, *Oscillospira*, and *Acidiphilium*, also exhibited segment-specific changes in response to DY201 supplementation.

Taken together, these results demonstrate that DY201 modulates the gut microbiota through a “fine-tuning” mechanism: it maintains overall community stability while selectively enriching beneficial bacteria and suppressing specific populations, accompanied by increased microbial diversity in the ileum. This pattern of precise modulation, rather than global restructuring, is characteristic of an ideal probiotic intervention.

## 3. Materials and Methods

### 3.1. Bacterial Isolation, Identification, and Environmental Tolerance Assays of Bacillus velezensis DY201

*Bacillus velezensis* strain DY201 was isolated by Shanxi Dayu Bioengineering Co., Ltd. (Yuncheng, China) from fecal samples of healthy broilers. Samples were subjected to serial dilution in sterile saline (0.85% NaCl), plated onto Luria–Bertani (LB) agar, and incubated at 37 °C for 24 h. Individual colonies exhibiting distinct morphologies were selected and purified through successive streaking. The isolate DY201 was chosen for further investigation due to its significant inhibitory activity observed in preliminary antimicrobial screenings.

Cellular morphology and Gram reaction were assessed using standard Hucker’s method, while endospore formation was evaluated via Schaeffer–Fulton staining. For ultrastructural analysis, exponential-phase cells were fixed with 2.5% glutaraldehyde, dehydrated through a graded ethanol series, critical-point-dried, and sputter-coated with a gold–palladium layer prior to imaging with a field-emission scanning electron microscope (JSM-IT700HR, JEOL Ltd., Tokyo, Japan).

Biochemical characteristics were profiled using the commercial HBIG14 *Bacillus* identification system (Hope Bio-Technology Co., Ltd., Qingdao, China) in accordance with the manufacturer’s protocols. Assays included Voges–Proskauer reaction, citrate utilization, starch hydrolysis, anaerobic growth, propionate utilization, gelatin liquefaction, nitrate reduction, and tolerance to 7% NaCl and pH 5.7.

The whole genome of DY201 was sequenced using a hybrid approach combining the Illumina NovaSeq 6000 platform for short-read data and the Oxford Nanopore PromethION platform for long-read sequencing (Guangdong Magigene Biotechnology Co., Ltd., Guangdong, China). De novo assembly was performed by integrating data from both platforms, resulting in a complete, gap-free genome sequence. ANI was calculated against the model strain *B. velezensis* FZB42 (GCF_000015785.2) to confirm taxonomic assignment. Furthermore, a whole-genome-based phylogenetic tree was reconstructed using the Type Strain Genome Server (TYGS). The complete genome sequence was deposited in the GenBank database under accession numbers CP182571 and CP182572.

Tolerance to environmental stresses was evaluated in five biological replicates. To assess pH tolerance, LB broth was adjusted to pH levels ranging from 2.0 to 11.0 (in 1.0-unit intervals) using sterile HCl or NaOH, inoculated with DY201 (initial inoculum ~10^6^ CFU/mL), and incubated at 37 °C. Thermotolerance was determined by monitoring growth in LB broth (pH 7.0) at 37 °C, 42 °C, and 50 °C. Gastrointestinal fluid tolerance was assessed by sequential exposure to ready-to-use commercial simulated gastric fluid (SGF; Coolaber, Beijing Coolaber Technology Co., Ltd., Beijing, China) and simulated intestinal fluid (SIF; Coolaber, Beijing Coolaber Technology Co., Ltd., Beijing, China). Both fluids were used directly according to the manufacturer’s instructions without additional modification or supplementation with exogenous digestive enzymes. Bacterial turbidity under these conditions was monitored by recording OD600 at 15 min intervals using a microbial growth curve analyzer (MGC-200, Ningbo Scientz Biotechnology Co., Ltd., Ningbo, China).

### 3.2. Assessment of In Vitro Antimicrobial Activity

Cell-free supernatant was prepared by centrifuging DY201 culture (24 h, 37 °C, 180 rpm) at 10,000× *g* for 10 min, followed by filtration through a 0.22 μm membrane.

Antimicrobial activity was evaluated using the agar well diffusion method against four indicator pathogens: ETEC K88 (CVCC 196), *S. aureus* (CVCC 1882), *S. pullorum* (CVCC 519), and *C. perfringens* (CVCC 66). Briefly, pathogen suspensions (10^6^ CFU/mL) were spread onto LB agar plates, and 100 μL of cell-free supernatant was added to 6 mm wells. Inhibition zone diameters were measured after 18–24 h incubation under appropriate atmospheric conditions. All experiments involving *C. perfringens* were conducted and incubated in an automated anaerobic chamber (YQXIII, Chuanhong Experimental Instrument Co., Ltd., Shanghai, China). For potency assessment, cell-free supernatant was serially diluted and tested similarly.

Thermal stability was assessed by incubating cell-free supernatant at 50 °C and 85 °C for 5 min prior to activity testing. Temperature-dependent activity was compared between cell-free supernatant from DY201 cultured at 37 °C and 42 °C. pH stability was evaluated using cell-free supernatant from DY201 cultured in LB broth adjusted to pH 5.0–8.0.

Gastrointestinal tolerance was tested by sequential exposure to SGF and SIF at 37 °C, followed by activity measurement.

### 3.3. Multi-Omics Analysis of Antimicrobial Mechanism

Indicator pathogens were incubated with DY201 cell-free supernatant for 20 min to 1.5 h, then fixed with 2.5% glutaraldehyde, dehydrated through a graded ethanol series, critical-point-dried, and sputter-coated with a gold–palladium layer prior to imaging with a field-emission scanning electron microscope (JSM-IT700HR, JEOL, Tokyo, Japan).

BGCs were identified using antiSMASH 7.0 by querying the DY201 genome against the Minimum Information about a Biosynthetic Gene Cluster (MIBiG) database. For multi-omics analysis, single colonies of DY201 were inoculated into six flasks each containing 100 mL of LB broth and cultured at 37 °C for 14 h with shaking at 250 rpm. From each culture, 30 mL of fermentation broth was centrifuged at 4000× *g* for 5 min to separate the supernatant and cell pellet. The cell pellets were then resuspended in SGF or SIF and incubated statically for an additional 4 h. After treatment, 30 mL of each suspension was centrifuged at 4000× *g* for 5 min to collect the supernatant and cell pellet separately. All samples were flash-frozen in liquid nitrogen and stored at −80 °C until further analysis. The cell pellets were subjected to proteomic analysis, and the supernatants were subjected to metabolomic analysis, both performed by Beijing Allwegene Technology Co., Ltd. (Beijing, China). For differential analysis between SGF-treated samples and SIF-treated samples, metabolites with |log_2_FC| > 2 and *p* < 0.05 were considered significantly upregulated. Metabolite identification was achieved by matching MS/MS spectra against public databases and the in-house database of Beijing Allwegene Technology Co., Ltd. (Beijing, China) and confirmed using authentic standards where available.

### 3.4. Animal Experiment and Sample Collection

One-day-old male Arbor Acres broilers (*n* = 16) were randomly assigned to two groups: a control group (basal diet) and a DY201 group. The DY201 group was fed the basal diet supplemented with DY201 fermentation broth preparation at 1 mg per kg of basal diet. The dosage refers to the mass of DY201 fermentation broth preparation added to the basal diet. The preparation was first premixed with a small amount of basal diet and then thoroughly incorporated into the complete diet. In this preliminary animal trial, the supplementation level was defined on a mass basis rather than standardized according to viable CFU per kg of feed. All experimental procedures were approved by the Institutional Animal Care and Use Committee of Northwest A&F University, China, and carried out in strict accordance with the ARRIVE guidelines and national animal welfare regulations. At day 28, all broilers were euthanized by cervical dislocation under anesthesia to minimize pain and distress. Luminal contents from the ileum and jejunum were aseptically collected for 16S rRNA gene sequencing.

### 3.5. 16S rRNA Gene Sequencing and Analysis

Total microbial genomic DNA was extracted from ileal and jejunal contents using the E.Z.N.A. Stool DNA Kit (Omega Bio-tek, Inc., Norcross, GA, USA) according to the manufacturer’s instructions. The V3-V4 hypervariable region of the 16S rRNA gene was amplified using the universal primers 338F and 806R. PCR products were purified and sequenced by Guangdong Magigene Biotechnology Co., Ltd. (Guangdong, China), on the Illumina MiSeq platform.

## 4. Discussion

In this study, we isolated and characterized the *Bacillus velezensis* strain DY201 from broiler feces and systematically evaluated its probiotic potential through integrated phenotypic, genomic, proteomic, metabolomic, and in vivo analyses. The strain exhibited potent broad-spectrum antimicrobial activity against key poultry pathogens, exceptional tolerance to gastrointestinal and feed-processing stresses, and segment-specific modulation of gut microbiota in broilers. Notably, multi-omics profiling revealed a dual antimicrobial strategy wherein DY201 employs small-molecule metabolites as primary effector molecules under the tested conditions, while maintaining lipopeptide biosynthetic machinery in a functionally poised state.

The taxonomic assignment of DY201 as *B. velezensis* was firmly established through morphological, biochemical, and genomic analyses, with the complete genome sequence and high ANI value (97.69%) relative to the type strain FZB42 providing definitive confirmation [13]. Beyond taxonomic confirmation, the strain exhibited robust growth across pH 4.0–9.0 and temperatures up to 50 °C, with optimal proliferation at 42 °C, a temperature that mirrors the core body temperature of broilers. This thermotolerance aligns with requirements for poultry probiotics, where host temperatures typically range from 40–43 °C. Furthermore, DY201 demonstrated 51.32% survival following sequential exposure to simulated gastric and intestinal fluids, exceeding tolerance levels reported for many *Lactobacillus* strains and comparable to other sporulated *Bacillus* probiotics, supporting its potential for effective intestinal delivery [21].

Building on these favorable phenotypic traits, we next evaluated the antimicrobial activity of DY201 against clinically relevant poultry pathogens. The strain exhibited potent inhibition against all four tested organisms: ETEC K88, *S. aureus*, *S. pullorum*, and *C. perfringens*, with activity persisting even after 1000-fold dilution, indicating high potency of secreted metabolites. This broad-spectrum activity is particularly relevant for poultry production, where co-infections and *C. perfringens*-associated necrotic enteritis impose significant economic burdens [22]. Moreover, the remarkable thermal stability (over 92.65% activity retained after 85 °C for 5 min) surpasses that of non-spore-forming probiotics and positions DY201 as suitable for incorporation into pelleted feeds [14]. Similarly, stable activity across pH 5.0–8.0 and substantial retention after simulated gastrointestinal exposure further support its oral application potential.

To elucidate the mechanistic basis of this antimicrobial activity, we employed an integrated multi-omics approach. Genomic analysis revealed a rich arsenal of BGCs encoding NRPS and PKS systems for lipopeptide production, including surfactin, iturin, fengycin, bacillomycin D, and the circular bacteriocin amylocyclicin. Notably, a 100% identical BGC for paenilarvin, which is an iturin-like lipopeptide previously reported mainly in *Paenibacillus larvae*, was identified, highlighting the unique metabolic potential of DY201. These findings initially suggested that lipopeptides might underlie the observed antimicrobial effects, consistent with the established view that *B. velezensis* strains are prolific antimicrobial lipopeptide producers [8].

However, proteomic and metabolomic analyses revealed an unexpected departure from this paradigm. While key biosynthetic enzymes for lipopeptide production were detected, confirming transcriptional and translational engagement of the corresponding gene clusters, the mature lipopeptide products themselves were conspicuously absent. Instead, differential metabolomic analysis comparing high-activity (SGF-treated) and low-activity (SIF-treated) samples identified distinct upregulated metabolites, including Withaferin A, 2’-Hydroxy-2-methoxychalcone, and Platycodigenin, as primary effectors associated with enhanced antimicrobial activity. These compounds are documented to disrupt bacterial membranes through lipid bilayer destabilization and pore formation, mechanisms fully consistent with the membrane perforation observed in our SEM analysis. This apparent discrepancy between genetic potential and metabolic output likely reflects the tight regulation of secondary metabolism in *Bacillus* [23], wherein under standard laboratory conditions, regulatory circuits governing NRPS/PKS expression may not be fully activated, resulting in sub-threshold lipopeptide production while the strain constitutively produces a distinct set of small molecules serving as immediate effectors. This “dual system” strategy, comprising constitutive small molecules and an inducible lipopeptide arsenal, may confer adaptive advantages, enabling rapid response to acute threats while reserving resource-intensive weapons for specific ecological contexts. The observed membrane-targeting mechanism of these small-molecule metabolites also provides a plausible explanation for the broad-spectrum activity observed across diverse pathogens, as membrane disruption represents a universal and evolutionarily difficult target for bacteria to overcome through resistance development.

Although mature lipopeptides were not confidently annotated in the non-targeted metabolomic dataset, this result should not be interpreted as definitive evidence for their complete absence. Cyclic lipopeptides such as surfactin, iturin, and fengycin are hydrophobic, structurally heterogeneous, and often occur as multiple homologues, and their detection is strongly influenced by extraction solvents, chromatographic conditions, ionization mode, ion suppression, matrix effects, and MS/MS database coverage. The non-targeted metabolomics workflow used in this study was designed for broad metabolite profiling rather than targeted lipopeptide quantification, and low-abundance lipopeptides may have remained below the detection or annotation threshold. Therefore, the present data indicate that several small-molecule metabolites were associated with the immediate antimicrobial activity observed under the tested conditions, but they do not exclude concomitant or inducible lipopeptide production. Future targeted LC-MS/MS analyses using authentic lipopeptide standards will be required to quantify surfactin-, iturin-, and fengycin-family compounds and to determine their precise contribution to the antimicrobial phenotype of DY201.

The relevance of these in vitro findings was further substantiated through in vivo evaluation in broilers. DY201 supplementation significantly modulated gut microbiota composition in a segment-specific manner, with the substantial increase in *Bacillus* abundance in both ileum (34-fold) and jejunum (7-fold) confirming successful intestinal delivery and validating the gastrointestinal tolerance observed in vitro. The segment-specific enrichment of *Lactobacillus* in the jejunum (73.05% to 80.11%) is particularly significant, as lactobacilli contribute to intestinal health through organic acid production, pathogen competition, and immunomodulation [24]. Conversely, the dramatic reduction of *Candidatus Arthromitus* in the ileum (13.38% to 0.59%; 95.6% decrease) warrants further investigation regarding its functional implications in poultry, though excessive abundance of this segmented filamentous bacterium has been associated with inflammatory responses in some contexts. Importantly, these compositional shifts occurred without significant alteration of overall community structure (β-diversity unchanged), indicating “fine-tuning” rather than global restructuring, a characteristic of ideal probiotic interventions that maintain ecosystem stability while selectively modulating key populations [25]. The increased α-diversity in the ileum further supports beneficial effects, as higher diversity is generally associated with enhanced ecosystem resilience and function.

Within the broader One Health framework, which recognizes the interconnectedness of human, animal, and environmental health, DY201 represents a promising antibiotic alternative that could reduce antimicrobial use in poultry production, thereby mitigating antimicrobial resistance selection and spread. Its broad-spectrum activity against zoonotic pathogens such as *S. pullorum* may also reduce foodborne transmission risks to humans. The strain’s exceptional environmental robustness and thermal stability further enhance its practical applicability in industrial feed processing.

Several limitations of this study should be acknowledged. First, the in vivo experiment included a relatively small number of broilers, with 16 birds in total and 8 birds per group, and did not include pen-level replication. Therefore, the microbiota-related findings should be interpreted as preliminary and hypothesis-generating rather than definitive evidence of flock-level efficacy. In addition, the in vivo evaluation was limited to intestinal microbiota composition and diversity. Functional intestinal health endpoints, including growth performance, intestinal morphology, epithelial barrier function, immune responses, and pathogen challenge outcomes, were not evaluated in the present study. Therefore, the observed microbiota changes should not be directly equated with confirmed intestinal health benefits. Larger, pen-replicated animal trials incorporating these functional endpoints are needed to determine whether DY201-mediated microbiota modulation translates into measurable improvements in broiler intestinal health and production performance. Second, the regulatory mechanisms governing the switch between constitutive small-molecule production and inducible lipopeptide synthesis remain to be elucidated, representing an important direction for future research.

In conclusion, the *Bacillus velezensis* strain DY201 combines robust environmental tolerance, broad-spectrum antimicrobial activity, and beneficial gut microbiota modulation, positioning it as a promising probiotic candidate for poultry production. The integrated multi-omics analysis revealed unexpected complexity in its antimicrobial strategy, challenging prevailing assumptions regarding lipopeptide dominance and highlighting the metabolic versatility of this species. These findings advance our understanding of Bacillus probiotics and provide a foundation for developing effective antibiotic alternatives within the One Health paradigm.

## 5. Conclusions

In this study, *Bacillus velezensis* DY201, isolated from broiler feces, was characterized as a candidate probiotic strain for poultry application. DY201 showed tolerance to a broad range of environmental conditions relevant to poultry production, including host body temperature, variable acidity, and simulated gastrointestinal exposure. Its cell-free fermentation supernatant exhibited broad-spectrum inhibitory activity against several important poultry-associated pathogens, including enterotoxigenic *Escherichia coli* K88, *Staphylococcus aureus*, *Salmonella pullorum*, and *Clostridium perfringens*. This activity was relatively stable under heat treatment, pH variation, and simulated digestive conditions, supporting its potential suitability for feed-related applications.

Integrated genomic, proteomic, and metabolomic analyses indicated that DY201 possesses multiple biosynthetic gene clusters associated with antimicrobial compound production. However, under the conditions tested in this study, the observed antimicrobial activity appeared to be more closely associated with small-molecule metabolites capable of damaging bacterial cell surfaces, rather than with mature lipopeptide products. Dietary supplementation with DY201 also modulated the gut microbiota of broilers in a segment-specific manner, increasing *Bacillus* abundance, enriching *Lactobacillus* in the jejunum, and reducing *Candidatus Arthromitus* in the ileum, without causing a major disruption of the overall microbial community structure. Overall, these findings suggest that *B. velezensis* DY201 is a promising probiotic candidate for supporting broiler intestinal health and may contribute to the development of antibiotic alternatives in poultry production. However, because the animal experiment involved only 16 broilers, with 8 birds per group and no pen-level replication, the in vivo microbiota findings should be regarded as preliminary. Further studies involving larger animal trials, growth performance evaluation, immune response analysis, and pathogen challenge models are needed to confirm its practical efficacy and safety.

## Data Availability

The complete genome sequence of *Bacillus velezensis* DY201 generated in this study is publicly available in the GenBank database of the National Center for Biotechnology Information (NCBI) under accession numbers CP182571 and CP182572. All other data supporting the findings of this study are available from the corresponding author upon reasonable request.

## Supplementary Materials

The following supporting information can be downloaded at https://www.mdpi.com/article/10.3390/ani16111677/s1, Table S1: Putative biosynthetic gene clusters (BGCs) identified in the genome of *Bacillus velezensis* strain DY201 and their similarity to known secondary metabolite clusters; Table S2: Effects of dietary *Bacillus velezensis* DY201 on the relative abundance of bacterial phyla in the ileum and jejunum of broilers at 28 days of age; Table S3: Effects of dietary *Bacillus velezensis* DY201 on the relative abundance of bacterial genera in the ileum and jejunum of broilers at 28 days of age.

## References

[1] Kogut M.H., Genovese K.J., Swaggerty C.L., He H., Broom L. (2018). Inflammatory phenotypes in the intestine of poultry: Not all inflammation is created equal. Poult. Sci..

[2] Khan S.H., Iqbal J. (2016). Recent advances in the role of organic acids in poultry nutrition. J. Appl. Anim. Res..

[3] Ge H., Fu S., Guo H., Hu M., Xu Z., Zhou X., Chen X., Jiao X. (2022). Application and challenge of bacteriophage in the food protection. Int. J. Food Microbiol..

[4] Golnari M., Bahrami N., Milanian Z., Rabbani Khorasgani M., Asadollahi M.A., Shafiei R., Fatemi S.S. (2024). Isolation and characterization of novel Bacillus strains with superior probiotic potential: Comparative analysis and safety evaluation. Sci. Rep..

[5] Abdel-Moneim A.E., Ali S.A.M., Sallam M.G., Elbaz A.M., Mesalam N.M., Mohamed Z.S., Abdelhady A.Y., Yang B., Elsadek M.F. (2024). Effects of cold-pressed wheat germ oil and Bacillus subtilis on growth performance, digestibility, immune status, intestinal microbial enumeration, and gene expression of broilers under heat stress. Poult. Sci..

[6] Khongthong S., Piewngam P., Roekngam N., Maliwan P., Kongpuckdee S., Jeenkeawpleam J., Rodjan P. (2025). Effects of dietary *Bacillus subtilis* 14823 on growth performance, gut barrier integrity and inflammatory response of broilers raised in a stressful tropical environment. Poult. Sci..

[7] Liu Y., Xiong M., Hu X., Li Y., Zhang W., He W., Luo S., Zang J., Yang W., Chen Y. (2024). Dietary Bacillus velezensis KNF-209 supplementation improves growth performance, enhances immunity, and promotes gut health in broilers. Poult. Sci..

[8] Fazle Rabbee M., Baek K.H. (2020). Antimicrobial Activities of Lipopeptides and Polyketides of Bacillus velezensis for Agricultural Applications. Molecules.

[9] Dunlap C.A., Kim S.J., Kwon S.W., Rooney A.P. (2016). Bacillus velezensis is not a later heterotypic synonym of Bacillus amyloliquefaciens; Bacillus methylotrophicus, Bacillus amyloliquefaciens subsp. plantarum and ‘Bacillus oryzicola’ are later heterotypic synonyms of Bacillus velezensis based on phylogenomics. Int. J. Syst. Evol. Microbiol..

[10] Lin X., Wang J., Hou Z., Ren S., Wang W., Yang Y., Yi Y., Zhang Y., Li R. (2024). Antifungal Potential and Mechanism of Bacillus velezensis HeN-7 Isolated from Tobacco Leaves on Bipolaris sorokiniana. Curr. Microbiol..

[11] Perini H.F., Pereira B.B., Sousa E.G., Matos B.S., Silva Prado L.C.D., Carvalho Azevedo V.A., Castro Soares S., Silva M.V.D. (2024). Inhibitory effect of Bacillus velezensis 1273 strain cell-free supernatant against developing and preformed biofilms of Staphylococcus aureus and MRSA. Microb. Pathog..

[12] Zhou J., Xie Y., Liao Y., Li X., Li Y., Li S., Ma X., Lei S., Lin F., Jiang W. (2022). Characterization of a Bacillus velezensis strain isolated from Bolbostemmatis Rhizoma displaying strong antagonistic activities against a variety of rice pathogens. Front Microbiol..

[13] Jain C., Rodriguez R.L., Phillippy A.M., Konstantinidis K.T., Aluru S. (2018). High throughput ANI analysis of 90K prokaryotic genomes reveals clear species boundaries. Nat. Commun..

[14] Casula G., Cutting S.M. (2002). Bacillus probiotics: Spore germination in the gastrointestinal tract. Appl. Environ. Microbiol..

[15] Usjak D., Dinic M., Novovic K., Ivkovic B., Filipovic N., Stevanovic M., Milenkovic M.T. (2021). Methoxy-Substituted Hydroxychalcone Reduces Biofilm Production, Adhesion and Surface Motility of Acinetobacter baumannii by Inhibiting ompA Gene Expression. Chem. Biodivers..

[16] Murugan R., Subramaniyan S., Priya S., Ragavendran C., Arasu M.V., Al-Dhabi N.A., Choi K.C., Guru A., Arockiaraj J. (2023). Bacterial clearance and anti-inflammatory effect of Withaferin A against human pathogen of Staphylococcus aureus in infected zebrafish. Aquat. Toxicol..

[17] Murugan R., Rajesh R., Seenivasan B., Haridevamuthu B., Sudhakaran G., Guru A., Rajagopal R., Kuppusamy P., Juliet A., Gopinath P. (2022). Withaferin A targets the membrane of Pseudomonas aeruginosa and mitigates the inflammation in zebrafish larvae; an in vitro and in vivo approach. Microb. Pathog..

[18] Rosselli S., Maggio A., Bellone G., Formisano C., Basile A., Cicala C., Alfieri A., Mascolo N., Bruno M. (2007). Antibacterial and anticoagulant activities of coumarins isolated from the flowers of Magydaris tomentosa. Planta Med..

[19] Abourashed E.A., Galal A.M., Shibl A.M. (2011). Antimycobacterial activity of ferutinin alone and in combination with antitubercular drugs against a rapidly growing surrogate of Mycobacterium tuberculosis. Nat. Prod. Res..

[20] Gilbert-Girard S., Reigada I., Savijoki K., Yli-Kauhaluoma J., Fallarero A. (2021). Screening of natural compounds identifies ferutinin as an antibacterial and anti-biofilm compound. Biofouling.

[21] Soylemez-Milli N., Erturkmen P., Alp Baltakesmez D. (2025). The Resistance Abilities of Some Bacillus Species to Gastrointestinal Tract Conditions: Whole Genome Sequencing of the Novel Candidate Probiotic Strains Bacillus clausiiBA8 and Bacillus subtilisBA11. Food Sci. Nutr..

[22] Van Immerseel F., De Buck J., Pasmans F., Huyghebaert G., Haesebrouck F., Ducatelle R. (2004). Clostridium perfringens in poultry: An emerging threat for animal and public health. Avian. Pathol..

[23] Lopez D., Vlamakis H., Kolter R. (2009). Generation of multiple cell types in Bacillus subtilis. FEMS Microbiol. Rev..

[24] Walter J. (2008). Ecological role of lactobacilli in the gastrointestinal tract: Implications for fundamental and biomedical research. Appl. Environ. Microbiol..

[25] Derrien M., van Hylckama Vlieg J.E. (2015). Fate, activity, and impact of ingested bacteria within the human gut microbiota. Trends Microbiol..

