# Supplementary material for: Characterization of Bacillus velezensis DY201: Antimicrobial Mechanisms and Intestinal Health Benefits in Broilers
Source: Animals (Basel). 2026 May 30;16(11):1677. doi: 10.3390/ani16111677 (PMC13255843; doi:10.3390/ani16111677)
Supplement: Supplementary file 1 [file animals-16-01677-s001.zip › animals-4328631-supplementary.pdf]

Supplementary Table S1. Putative biosynthetic gene clusters (BGCs) identified in the genome of *Bacillus velezensis* strain DY201 and their similarity to known secondary metabolite clusters.

MIBiG accession	Metabolites	Region	Synthesis type	Similarity
BGC0000433	Surfactin	Region1.1	NRP:Lipopeptide	82%
BGC0001383	Macrolactin H<br>Macrolactin B<br>Macrolactin 1c<br>Macrolactin E	Region1.6	Polyketide	100%
BGC0001089	Bacillaene	Region1.7	Polyketide+NRP	100%
BGC0001095	Fengycin	Region1.8	NRP	100%
BGC0000407	Plipastatin	Region1.8	NRP	100%
BGC0001090	Bacillomycin D	Region1.8	Polyketide+NRP:Lipopeptide	100%
BGC0001103	Mycosubtilin	Region1.8	NRP+Polyketide	100%
BGC0001098	Iturin	Region1.8	NRP+Polyketide	88%
BGC0000402	Paenilarvin A<br>Paenilarvin B<br>Ppaenilarvin C	Region1.8	NRP	100%
BGC0000176	Difficidin	Region1.11	Polyketide	100%
BGC0000401	Paenibactin	Region1.12	NRP	100%
BGC0001185	Bacillibactin	Region1.12	NRP siderophore	100%
BGC0000616	Amylocyclicin	Region1.12	RiPP:Head-to-tailcyclized peptide	100%
BGC0001184	Bacilysin	Region1.13	Other	100%

Supplementary Table S2. Effects of dietary Bacillus velezensis DY201 on the relative abundance of bacterial phyla in the ileum and jejunum of broilers at 28 days of age.

Phylum	Ileum Control (%)	Ileum DY201 (%)	Jejunum Control (%)	Jejunum DY201 (%)
Firmicutes	87.50470882	84.31486082	82.90339586	88.58780216
Proteobacteria	9.609592885	13.07842965	15.07555623	11.03927486
Cyanobacteria	2.216940373	2.067515874	0.532596807	0.145147507
Tenericutes	0.641078265	0.408379826	0.73854382	0.069973893
Actinobacteria	0.017241519	0.018143942	0.257127436	0.077416633
Bacteroidetes	2.85E-03	0.08131835	0.159068283	0.071322328
Chloroflexi	8.56E-05	5.26E-04	0.081884267	0
[Thermi]	7.13E-04	2.44E-03	0.050483502	0
Other	6.79E-03	0.028383127	0.2013438	9.06E-03

Supplementary Table S3. Effects of dietary Bacillus velezensis DY201 on the relative abundance of bacterial genera in the ileum and jejunum of broilers at 28 days of age.

Genus	Ileum-Control (%)	Ileum-DY201 (%)	Jejunum-Control (%)	Jejunum-DY201 (%)
Lactobacillus	63.53860432	59.39977425	73.04563133	80.10664346
Bacillus	0.299961763	10.30155499	0.76686509	5.564342991
Candidatus Arthromitus	13.3819905	0.586205823	0.050816049	0.035829378
Ruminococcus	5.01251504	5.195985927	0.784906572	0.61011508
[Ruminococcus]	1.94618924	3.670195163	3.520044279	1.412252603
Vibrio	0.093561331	0.065509368	4.19461833	5.512110483
Oscillospira	4.792702626	3.495545787	1.419287016	0.382072773
Acidiphilium	0.46413503	1.007685125	6.30796018	0.908936712
Other	10.47034015	16.27754356	9.909871156	5.467696519
